# Central administration of dipeptides, beta-alanyl-BCAAs, induces hyperactivity in chicks

**DOI:** 10.1186/1471-2202-8-37

**Published:** 2007-05-31

**Authors:** Yousuke Tsuneyoshi, Shozo Tomonaga, Mari Asechi, Koji Morishita, D Michael Denbow, Mitsuhiro Furuse

**Affiliations:** 1 Laboratory of Advanced Animal and Marine Bioresources, Graduate School of Bioresource and Bioenvironmental Sciences, Kyushu University, Fukuoka 812-8581, Japan; 2 Healthcare Product Development Center, Kyowa Hakko Kogyo Co., Ltd, Tsukuba 305-0841, Japan; 3 Department of Animal and Poultry Sciences, Virginia Polytechnic Institute and State University, Blacksburg, VA 24061-0306, USA

## Abstract

**Background:**

Carnosine (β-alanyl-L-histidine) is a putative neurotransmitter and has a possible role in neuron-glia cell interactions. Previously, we reported that carnosine induced hyperactivity in chicks when intracerebroventricularly (i.c.v.) administered. In the present study, we focused on other β-alanyl dipeptides to determine if they have novel functions.

**Results:**

In Experiment 1, i.c.v. injection of β-alanyl-L-leucine, but not β-alanyl-glycine, induced hyperactivity behavior as observed with carnosine. Both carnosine and β-alanyl-L-leucine stimulated corticosterone release. Thus, dipeptides of β-alanyl-branched chain amino acids were compared in Experiment 2. The i.c.v. injection of β-alanyl-L-isoleucine caused a similar response as β-alanyl-L-leucine, but β-alanyl-L-valine was somewhat less effective than the other two dipeptides. β-Alanyl-L-leucine strongly stimulated, and the other two dipeptides tended to stimulate, corticosterone release.

**Conclusion:**

These results suggest that central β-alanyl-branched chain amino acid stimulates activity in chicks through the hypothalamus-pituitary-adrenal axis. We named β-alanyl-L-leucine, β-alanyl-L-isoleucine and β-alanyl-L-valine as Excitin-1, Excitin-2 and Excitin-3, respectively.

## Background

β-Alanine is the only naturally occurring β-amino acid found in its free state in the brain. It is a component of carnosine (β-alanyl-L-histidine) and anserine (β-alanyl-1-methyl-L-histidine). Carnosine, as well as its related compounds anserine and homocarnosine, were discovered in skeletal muscles and nervous tissues [1,2]. Ever since carnosine was discovered, it is thought to be a putative neurotransmitter in the olfactory receptor neurons [3]. Biochemically, carnosine seems to possess a number of protective functions including anti-oxidant, free-radical scavenger and anti-glycating agent, as well as being able to bind to protein carbonyls and suppress their cross-linking activity [4,5].

However, the functions of carnosine and anserine in the central nervous system (CNS) are still obscure. Recently, intracerebroventricular (i.c.v.) injection of carnosine dramatically induced hyperactivity such as increasing spontaneous activity and distress vocalizations and increasing plasma corticosterone concentration in chicks [6]. Part of this function occurred through nitric oxide (NO) production via carnosine administration [7].

β-Alanine is synthesized in the liver as the final metabolite of uracil and thymin degradation [8]. It was reported that β-alanine decreased the toxic effects of β-amyloid in rat brain endothelial cells [9] or reduced bacterial lipopolysaccharide-induced hepatotoxicity in rats [10]. Although β-alanine is a non-proteinaceous amino acid, it is especially rich in the brain and the breast muscle of chickens as components of carnosine or anserine, or as a free amino acid [11], therefore it is reasonable to speculate that β-alanine may have other functions. Accordingly, we focused on its role as a neurotransmitter and investigated the effect of dipeptides having β-alanine at the amino terminus. In a general way, the discovery of the novel dipeptides has been done through the process of isolation and purification, but we designed and manufactured dipeptides related to carnosine, and screened these dipeptides with special reference to their effects on behavior.

In Experiment 1, we investigated effects of β-alanyl-L-leucine (β-Ala-L-Leu), β-alanyl-glycine (β-Ala-Gly) and carnosine on behavior and effect of β-Ala-L-Leu was compared to carnosine. Then we studied dipeptides involved in branched chain amino acids (BCAAs) at the carboxyl terminus in Experiment 2, namely β-alanyl-L-leucine (β-Ala-L-Leu), β-alanyl-L-isoleucine (β-Ala-L-Ile) and β-alanyl-L-valine (β-Ala-L-Val).

## Results

**Figure 1:**
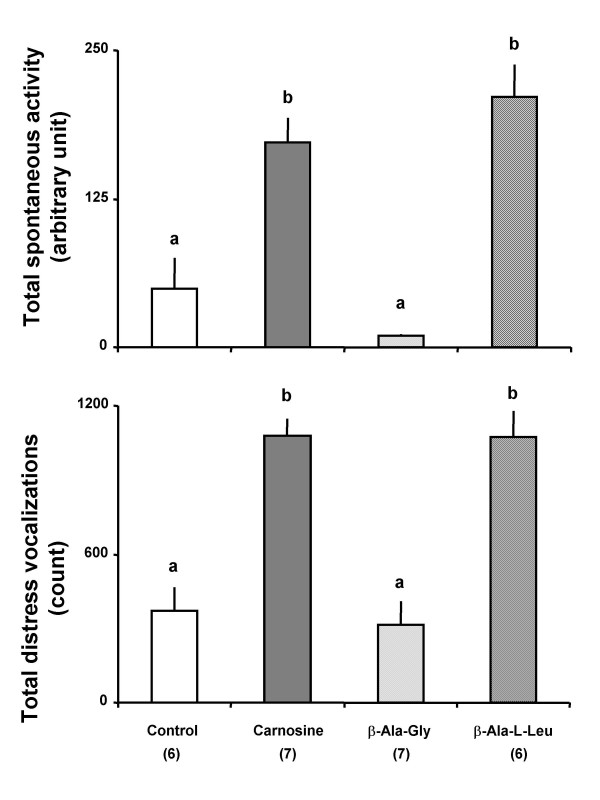
Effect of i.c.v. injections of carnosine, β-Ala-Gly and β-Ala-L-Leu on total spontaneous activity (upper panel) and total distress vocalizations (lower panel) in chicks for 10 min postinjection. Figures in parentheses indicate the number of chicks in each group. Results are expressed as means ± S.E.M. Groups with different letters are significantly different (P < 0.05).

**Figure 2:**
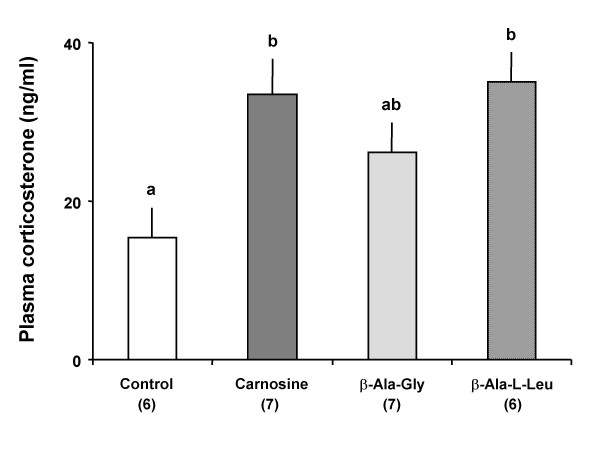
Effect of i.c.v. injections of carnosine, β-Ala-Gly and β-Ala-L-Leu on plasma corticosterone concentration after a 10 min behavioral analysis in chicks. Figures in parentheses indicate the number of chicks in each group. Results are expressed as means ± S.E.M. Groups with different letters are significantly different (P < 0.05).

Figure 1 shows the effect of i.c.v. injection of dipeptides on total spontaneous activity (upper panel) and distress vocalizations (lower panel) during the 10 min isolation (Experiment 1). Significant effects on total spontaneous activity (F(3, 22) = 21.352, P < 0.0001) and total distress vocalizations (F(3, 22) = 22.125, P < 0.0001) were detected. Carnosine and β-Ala-L-Leu significantly enhanced both parameters, but the effect of β-Ala-Gly was comparable to that of the control. The effect of i.c.v. injection of dipeptides on plasma corticosterone concentration after the behavioral tests is shown in Figure 2. Significant effects (F(3, 22) = 4.792, P < 0.0001) were observed. Carnosine and β-Ala-L-Leu significantly, and β-Ala-Gly tended to increase plasma corticosterone concentration.

Table 1 shows the effect of i.c.v. injection of carnosine, β-Ala-Gly and β-Ala-L-Leu on behavior during 10-min observation period. The chicks receiving carnosine and β-Ala-L-Leu tended to be active, but not significantly more than those receiving saline. A significant effect was detected in standing/sitting motionless with eyes opened (F(3, 22) = 3.332, P < 0.05), with the chicks receiving for carnosine being lowest and those receiving β-Ala-Gly the highest.

**Figure 3:**
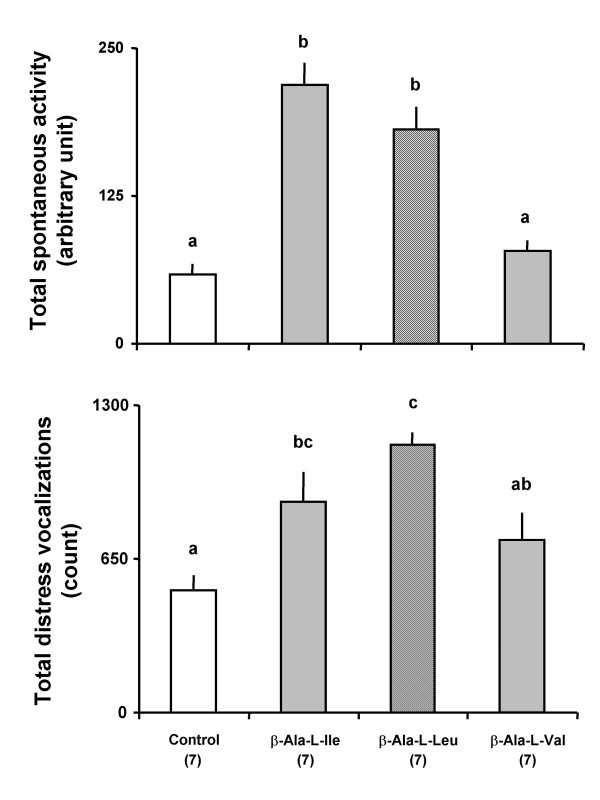
Effect of i.c.v. injections of β-Ala-L-Ile, β-Ala-L-Leu and β-Ala-L-Val on total spontaneous activity (upper panel) and total distress vocalization (lower panel) in chicks for 10 min postinjection. Figures in parentheses indicate the number of chicks in each group. Results are expressed as means ± S.E.M. Groups with different letters are significantly different (P < 0.05).

**Figure 4:**
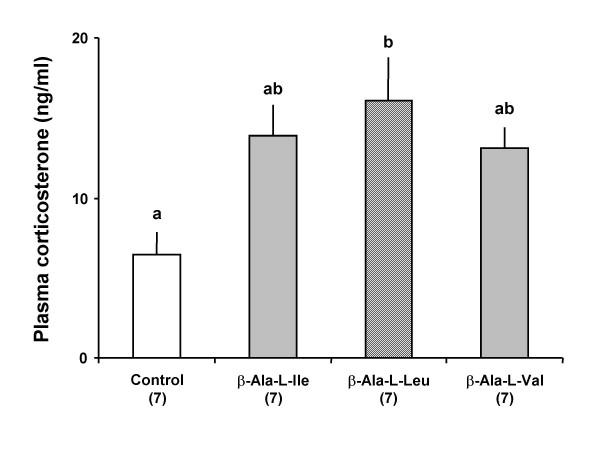
Effect of i.c.v. injections of β-Ala-L-Ile, β-Ala-L-Leu and β-Ala-L-Val on plasma corticosterone level after a 10 min behavioral analysis in chicks. Figures in parentheses indicate the number of chicks in each group. Results are expressed as means ± S.E.M. Groups with different letters are significantly different (P < 0.05).

Figure 3 shows the effect of i.c.v. injection of β-alanyl dipeptides having BCAAs at the carboxyl terminus on total spontaneous activity (upper panel) and total distress vocalizations (lower panel) during the 10 min isolation (Experiment 2). Significant effects on total spontaneous activity (F(3, 24) = 27.786, P < 0.0001) and total distress vocalizations (F(3, 24) = 7.528, P < 0.01) were detected. For the spontaneous activity, β-Ala-L-Ile has the greatest effect, followed by β-Ala-L-Leu and β-Ala-L-Val. For the distress vocalizations, on the other hand, the order of the effectiveness was β-Ala-L-Leu, β-Ala-L-Ile and β-Ala-L-Val. Figure 4 shows the effect of i.c.v. injection of dipeptides involved in BCAAs on plasma corticosterone concentration after the behavioral tests, and significant effects (F(3.24) = 4.747, P < 0.001) were found. β-Ala-L-Leu significantly enhanced, and both β-Ala-L-Ile and β-Ala-L-Gly tended to increase, plasma corticosterone concentration.

The effect of i.c.v. injection of β-Ala-L-Ile, β-Ala-L-Leu and β-Ala-L-Val on behavioral categories of chicks during 10 min behavior observation is shown in Table 2. Significant effects were detected in active wakefulness (F(3, 24) = 6.209, P < 0.01) and standing/sitting motionless with eyes opened (F(3, 24) = 13.303, P < 0.0001). Compared with the control, injections of β-Ala-L-Ile, β-Ala-L-Leu and β-Ala-L-Val significantly increased the time for active wakefulness, but the reverse was true for standing/sitting motionless with eyes opened.

## Discussion

In Experiment 1, i.c.v. injection of β-Ala-L-Leu induced hyperactivity, enhanced distress vocalizations and increased plasma corticosterone concentration, and these effects were similar to those of carnosine. The effect of carnosine was consistent with the previous report [6]. Although the detailed mechanism is not known, β-Ala-L-Leu and carnosine may have a similar function in the central nervous system. The effect of other dipeptides (β-Ala-L-Ala, β-Ala-β-Ala and β-Ala-L-Lys) has been investigated, but no effect was detected (date not shown). BCAAs, i.e., L-leucine, L-isoleucine and L-valine have a similar structure. We thought that the effects of β-alanyl dipeptides having BCAAs at the carboxyl terminus might have a similar function. Thus, we further investigated the effect of β-Ala-L-Leu, β-Ala-L-Ile and β-Ala-L-Val in Experiment 2.

The i.c.v. injection of β-Ala-L-Ile had a similar effect for hyperactivity, distress vocalizations and plasma corticosterone concentration as observed with carnosine (Experiment 1) and β-Ala-L-Leu (Experiments 1 and 2). On the other hand, the effect of β-Ala-L-Val was somewhat weaker than that of β-Ala-L-Leu and β-Ala-L-Ile. At present, although we could not clarify the difference in efficacies among the three β-alanyl-BCAAs, the low molecular weight of L-valine may be associated with its weak activity. The sedative and hypnotic postures such as standing motionless with eyes closed and sleeping posture were rarely observed with the dipeptides applied here (Tables 1 and 2). It was concluded that one of the central functions in β-alanyl-BCAAs is induction of hyperactivity.

The mechanisms by which β-Ala-L-Leu and β-Ala-L-Ile function in the brain were unclear. The possibility that the hyperactivity effect is induced by individual constitutive amino acids is low. Although BCAAs are able to influence neurotransmitter levels in the brain with effects on behavior and stimulated food intake, especially L-leucine [12], the acute degradation of dipeptides could not occur in the brain. In fact, i.c.v. injection of β-alanine (0.8 μmol) as a constituent of carnosine decreased active wakefulness and increased sleeping posture [6]. The function of β-alanine was the reverse from that of carnosine. For these reasons, we considered the dipeptidyl structure as important for induction of hyperactivity.

In Experiments 1 and 2, dipeptides increased plasma corticosterone concentration. Corticosterone is released from the adrenal glands under the control of the hypothalamic pituitary adrenal (HPA) axis, and the activation of the HPA axis by acute stress produces a transient increase in plasma corticosterone levels in birds. Feltenstein et al. [13] demonstrated that a positive relationship exists between plasma corticosterone level and the behavioral response. Further, the response of layer-type neonatal chicks is more sensitive than meat-type [14]. β-Alanyl dipeptides applied here are suggested as stimulants for the HPA axis.

Thomas [15] suggested that drugs having activity to enhance production of NO such as carnosine would have beneficial effects in the treatment of neurodegenerative diseases. It was speculated that central carnosine might regulate brain function and/or behaviors by NO generation via cNOS in chicks [7]. On the other hand, Mrowiec et al. [16] showed that i.c.v. injection of L-phenylalanyl-L-arginine dipeptides increased the locomotor activity in mice. This effect was possibly associated with the modulation of the opiate receptor functions in the brain since the effect was evidently diminished by naloxone, and intrathecal injections of this peptide are ineffective in changing locomotor activity. Further experiments will be needed to clarify the precise mechanism of hyperactivity in the brain.

## Conclusion

We focused on a role of β-alanine as a neurotransmitter and investigated the effect of dipeptides having β-alanine at the amino terminus. In Experiment 1 and 2, we investigated the effect of i.c.v. injection of some dipeptides. Then, β-alanyl-branched chain amino acids, i.e., β-Ala-L-Leu, β-Ala-L-Ile and β-Ala-L-Val, induced hyperactivity behavior as observed with carnosine. β-Ala-L-Leu strongly stimulated, and the other two depeptides tended to stimulate, corticosterone release. These results suggests that central β-alanyl-branched chain amino acid, stimulates activity in chicks through the hypothalamus-pituitary-adrenal axis. These peptides may be useful drugs for the excitement, but we did not confirme whther these dipeptides are present in the brain. Further studies remain to be done. Finally we propose that β-Ala-L-Leu be named as Excitin-1, β-Ala-L-Ile as Excitin-2 and β-Ala-L-Val as Excitin-3 through their functions in excitements.

## Methods

### Animals and food

Day-old male chicks (Julia strain) were purchased from a local hatchery (Murata Hatchery, Fukuoka, Japan). The chicks were maintained in a windowless room at constant temperature 30 ± 1°C and continuous lighting. Food (Toyohashi Feed and Mills Co. Ltd., Aichi, Japan) and water were freely accessible. The chicks were raised in a group (20–25 per cage) until being placed on experiment. On the day of the experiment, the chicks (5- or 6-old-day) were distributed into experimental groups based on their body weight so that the average body weight of each group was as uniform as possible. Experimental procedures followed the guides for animal experiments in the Faculty of Agriculture and the Graduate Course of Kyushu University, as well as the Law (No. 105) and Notification (No. 6) of the Government.

### Preparation of drugs

Carnosine, β-Ala-L-Leu, β-Ala-Gly, β-Ala-L-Ile and β-Ala-L-Val were gifted from Kyowa Hakko Kogyo (Tokyo, Japan). Drugs were dissolved in 0.85% saline containing 0.1% Evans Blue solution and control groups were given the saline solution. At the end of the experiment, the birds were sacrificed with an overdose of sodium pentobarbital after which the location of the injection was verified. Data from individuals not having Evans Blue dye present in the lateral ventricle were deleted.

### Experimental procedure

The i.c.v. injections were in a volume of 10 μl using a microsyringe according to the method of Davis et al. [17]. In Experiment 1, chicks were divided into four groups, and given i.c.v. injections of saline as a control and 2.8 μmol of carnosine, β-Ala-L-Leu or β-Ala-Gly. In Experiment 2, chicks were given i.c.v. injections of saline as a control and 2.8 μmol of β-Ala-L-Leu, β-Ala-L-Ile and β-Ala-L-Val. The molarity and volume applied was determined by previous study [6]. We used an acrylic device to hold the head of chicks. A head holder with a hole in the head plate accommodated the 26-gauge needle of a Hamilton which was microsyringe directed into the lateral ventricle was used to make i.c.v. injections. The injection depth was approximately 0.6 cm from the bottom of the head plate. After the injection, chicks were immediately placed in an acrylic monitoring cage (W40 cm × D30 cm × H20 cm), and behavioral observations were made for 10 min. The monitoring systems were set in a separate room to avoid disturbing the animals. Spontaneous activity was automatically determined by utilizing infrared beam sensors (Neuroscience Inc., Tokyo, Japan) placed above the center of the monitoring cage and analyzed by the software DAS-008 (Neuroscience Inc.). The number of distress vocalizations was simultaneously recorded and counted, using a computer with Gretchen software (Excla, Inc.). Chicks were recorded by three video cameras positioned in different directions. Based on the method by van Luijtelaar et al. [18], the behaviors were classified into four categories: (1) active wakefulness; (2) standing/sitting motionless with eyes opened; (3) standing motionless with eyes closed; and (4) sitting motionless with head drooped (sleeping posture) by watching the videotapes. A direct correlation exists between sleeping posture and electrophysiological sleep with EEG measurement [18]. During the monitoring period, chicks were not given food or water.

### Plasma corticosterone analysis

Blood was collected in heparinized microtubes from the jugular vein immediately after the behavioral tests. In addition, the intact groups (with neither i.c.v. injection nor isolation stress) were also monitored in each experiment. Blood was centrifuged at 4°C at 10,000 × g for 4 min, and plasma was collected and stored at -30°C until analysis. Plasma corticosterone concentration was determined using a corticosterone enzyme immunoassay kit (Assay Designs Inc., MI, USA).

### Statistical analysis

Data were statistically analyzed by one-way analysis of variance (ANOVA) and a Tukey-Kramer test was done as a post hoc test. Significant differences implied P < 0.05. Values are presented as means ± S.E.M. Statistical analysis was made using commercially available package, Stat View (Version 5, SAS institute, Cary, USA, 1998).

## Authors' contributions

YT carried out the majority of the experiments and wrote a first draft of the manuscript, ST and MA advised and improved the methods based on their previous or preliminary experiments, and participated in partly experiments, KM assisted the preparation of experiments and helped to draft the manuscript, DMD advised and revised the draft manuscript, and wrote the final manuscript, MF conceived the study, participated in its design and coordination of the study, and helped to draft the manuscript. All authors read and approved the final manuscript.

## Figures and Tables

**Table 1:** Influence of i.c.v. injections of carnosine, β-Ala-Gly and β-Ala-L-Leu on various behavioral categories of chicks.

	Saline (6)	Carnosine (7)	β-Ala-Gly (7)	β-Ala-L-Leu (6)
Active wakefulness	345 ± 102	579 ± 4	402 ± 72	545 ± 20
Standing/sitting motionless with eyes open	111 ± 52 ab	21 ± 4 a	166 ± 46 b	55 ± 20 ab
Standing motionless with eyes closed	0	0	0	0
Sitting motionless with head drooped (sleeping posture)	144 ± 94	0	32 ± 28	0

Total (second)	600	600	600	600

Values are means ± S.E.M. in seconds. Figures in parentheses indicate the number of chicks in each group. Groups with different letters are significantly different (P < 0.05).

**Table 2:** Influence of i.c.v. injections of β-Ala-L-Ile, β-Ala-L-Leu and β-Ala-L-Val on various behavioral categories of chicks.

	Saline (7)	β-Ala-L-Ile (7)	β-Ala-L-Leu (7)	β-Ala-L-Val (7)
Active wakefulness	458 ± 49 a	575 ± 10 b	589 ± 6 b	584 ± 6 b
Standing/sitting motionless with eyes open	70 ± 7 a	25 ± 10 b	11 ± 6 b	16 ± 6 b
Standing motionless with eyes closed	15 ± 15	0	0	0
Sitting motionless with head drooped (sleeping posture)	57 ± 37	0	0	0

Total (second)	600	600	600	600

Values are means ± S.E.M. in seconds. Figures in parentheses indicate the number of chicks in each group. Groups with different letters are significantly different (P < 0.05).
